# Noradrenergic and Dopaminergic modulation of meta-cognition and meta-control

**DOI:** 10.1371/journal.pcbi.1012675

**Published:** 2025-02-26

**Authors:** Sara Ershadmanesh, Sahar Rajabi, Reza Rostami, Rani Moran, Peter Dayan

**Affiliations:** 1 Department of Computational Neuroscience, MPI for Biological Cybernetics, Tuebingen, Germany; 2 Cognitive Systems Laboratory, School of Electrical and Computer Engineering, University of Tehran, Tehran, Iran; 3 Department of Psychology, University of Tehran, Tehran, Iran; 4 Max Planck/UCL Centre for Computational Psychiatry and Ageing Research, London, United Kingdom; 5 Queen Mary University of London, London, United Kingdom; 6 Eberhard Karls University of Tübingen, Tübingen, Germany; Dartmouth College Department of Psychological and Brain Sciences, UNITED STATES OF AMERICA

## Abstract

Humans and animals use multiple control systems for decision-making. This involvement is subject to meta-cognitive regulation – as a form of control over control or meta-control. However, the nature of this meta-control is unclear. For instance, Model-based (MB) control may be boosted when decision-makers generally lack confidence as it is more statistically efficient; or it may be suppressed, since the MB controller can correctly assess its own unreliability. Since control and metacontrol are themselves subject to the influence of neuromodulators, we examined the effects of perturbing the noradrenergic (NE) and dopaminergic (DA) systems with propranolol and L-DOPA, respectively. We first administered a simple perceptual task to examine the effects of the manipulations on meta-cognitive ability. Using Bayesian analyses, we found that 81*%* of group M-ratio samples were lower under propranolol relative to placebo, suggesting a decrease of meta-cognitive ability; and 60*%* of group M-ratio samples were higher under L-DOPA relative to placebo, considered as no effect of L-DOPA on meta-cognitive ability . We then asked subjects to provide choices and confidence ratings in a two-outcome decision-making task that has been used to dissociate Model-free (MF) and MB control. MB behavior was enhanced by propranolol, while MF behavior was not significantly affected by either drug. The interaction between confidence and MF/MB behavior was highly variable under propranolol, but under L-DOPA, the interaction was significantly lower/higher relative to placebo. Our results suggest a decrease in metacognitive ability under the influence of propranolol and an enhancement of MB behavior and meta-control under the influence of propranolol and L-DOPA, respectively. These findings shed light on the role of NE and DA in different aspects of control and meta-control and suggest potential avenues for mitigating dysfunction.

## Author summary

Humans and other animals make decisions using different neural systems. Their operation and relative deployment is monitored and regulated through the function of what is known as meta-cognition. However, how meta-cognition works is not fully understood. We therefore used drugs in healthy human volunteers to manipulate the workings of two neurotransmitters, norepinephrine (NE) and dopamine (DA) that are believed to influence decision-making and meta-cognition. We first tested how these manipulations affected people’s ability to evaluate their own performance in simple tasks. We found that interfering with NE decreased this self-evaluation ability, while increasing DA did not have a significant effect. We then looked at their influence over decision-making in a more complex task designed to distinguish between reflexive or habitual and reflective or goal-directed control systems. We found that the NE manipulation boosted reflective planning, but did not affect habitual behavior. Conversely, increasing DA increased people’s confidence in their planning abilities. Our findings suggest that NE and DA play crucial roles in how we monitor and control our actions, with potential implications for treating neurological and psychiatric conditions in which these processes are disrupted.

## 1. Introduction

Humans and other animals exhibit an exquisite ability to adapt their information processing flexibly to the demands of decision-making tasks. In general, this requires us to have a good sense of the current state of ongoing computations, and then a means of regulating future operations accordingly. These capacities are sometimes referred to as forms of metaprocessing – concerned with monitoring (known generically as forms of metacognition), and regulation (forms of metacontrol). Individuals differ greatly in their metaprocessing capabilities, and impairments have been associated with psychiatric disorders such as schizophrenia, attention-deficit hyperactivity disorder, and obsessive-compulsive symptoms [1,2].

Although the concept is more general, most work on metacognitive sensitivity concerns *confidence,* i.e., the certainty we have about the correctness of our choices. To be appropriately metacognitively sensitive is to have high confidence in correct choices and low confidence in incorrect ones, a characteristic that has been variously quantified, particularly in perceptual decision-making tasks [3,4]. Note, though, that metacognitive ability is not fully determined by metacognitive sensitivity, since it is easy to be rightfully confident about the rectitude of easy choices. It is therefore necessary to correct for the difficulty of the decision-making task to quantify metacognitive ability. This quantity is then often referred to as metacognitive efficiency.

There is also substantial work on metacontrol as a whole – often under the rubric of cognitive control [5]. However, there are rather fewer studies that put together metacognition and metacontrol such that, for instance, after making a decision with low, rather than high, confidence, individuals might explore more options or seek additional information [6,7]. This combination is our focus.

One of the most important collection of neural substrates associated with these forms of meta-processing are neuromodulators, including norepinephrine (NE), dopamine (DA), acetylecholine (ACh), and serotonin (5-HT). These have long been associated with representing information essential to meta-processing such as different forms of uncertainty [8], and also in regulating diverse aspects of neural processing by changing excitability and plasticity of other neural elements [9–16] as would be required for meta-control. Furthermore, neuromodulators are deeply implicated in a wide variety of psychiatric conditions and also therapeutic approaches to these conditions [17–20]. Among the neuromodulators mentioned, we focused on norepinephrine (NE) and dopamine (DA) due to their prominent roles in modulating metacognition and metacontrol [21–23].

There has thus been some investigation of the role of particular neuromodulators in meta-processing. For instance, in a previous study by Hauser et al. [22], the role of NE and DA in perceptual meta-cognition was examined. The authors found enhanced meta-cognitive efficiency in a perceptual task using a $\beta_{2}$ inhibitor, propranolol, which is believed to weaken the effects of NE [13]. However, meta-cognitive efficiency was not affected by the blockade of dopamine D2/3 receptors with amisulpride. By contrast, Clos et al. [24] observed impairment of meta-cognition in memory discipline under the influence of the dopamine D2 antagonist haloperidol. Thus, the manipulation of NE and DA neuromodulators seems to be an important candidate for studying the modulation of metacognition.

In terms of meta-control, a recently fecund area which has been extensively explored is that of the balance between goal-directed/reflective/model-based (MB) and habitual/reflexive/model-free (MF) learning and planning [25–27]. These systems occupy opposite positions on spectra of computational complexity (MB being more taxing than MF, including demands on working memory); and statistical efficiency (and so flexibility/accuracy; MB besting MF). Thus, many factors have been suggested as influencing arbitration between these systems, including the diversity of rewards, stress, ambient reward rates, task difficulty, and training time [28–30], many of which can affect, or be affected by, neuromodulatory systems.

There are also indications of roles for NE and DA on meta-control. There is the general observation that NE helps mediate the balance between reflexive and reflective behavior [13]. Stress, which is associated with higher NE, has been found to impair specifically pre-frontal functions [31,32]; with MB behavior being particularly affected in individuals with lower working memory capacity [28]. Also, Wunderlich et al. [23] observed that higher DA levels induced by L-DOPA (Levodopa) were associated with increased MB behavior in the popular two-step task [33], putatively via DA’s positive effects on working memory [34,35]. By contrast, in a different and arguably simpler, two-outcome task [27], Deserno et al. [21] found that higher levels of dopamine, also achieved through L-DOPA administration, resulted in enhanced cooperation between MB and MF systems (improved guidance of MF credit assignment by MB inference) without a corresponding increase in MB behavior. In the two-outcome task used by Deserno et al. [21], there were also some trials called ’uncertainty,’ where subjects had to infer the chosen option by a ghost according to the learned task structure. Thus, the influence of MB inference on MF behavior could be studied.

However, we noted above that meta-control should depend on meta-cognition, for instance with confidence in the predictions of MF and MB systems helping determine their relative dominance [25]. Since these previous studies into meta-control did not assess meta-cognition, it is not clear whether the effects of L-DOPA operated directly on MF/MB control systems or indirectly via meta-control over MF/MB systems. Similarly, it is not known whether the conventional finding that meta-cognitive efficiency and average confidence are correlated across different tasks [36–38] would extend to domain-general effects of pharmacological manipulations. Thus, the main question of this study is about the pharmacological manipulation of meta-cognition and meta-control across two domains.

We therefore manipulated NE and DA pharmacologically using propranolol and L-DOPA in healthy volunteers, and examined the consequences for perceptual meta-cognition [36] (as a replication of Hauser et al. [22], and for comparison) and both meta-cognition and meta-control in a novel version of the two-outcome task in which we could examine explicitly the interaction between confidence and arbitration. Under propranolol, 81*%* of the group M-ratio samples were lower compared to the placebo condition in the perceptual task, and the interaction between confidence and MB behavior in the two-outcome task was highly variable. Under L-DOPA, only 60*%* of group M-ratio samples were higher than those in the placebo condition during the perceptual task, and the interaction between confidence and MF/MB control in the two-outcome task was lower/greater. In summary, our results suggest a diminution of metacognitive ability and an enhancement of MB computations by propranolol, and an improvement in meta-control under L-DOPA.

## 2. Results

**Fig 1:**
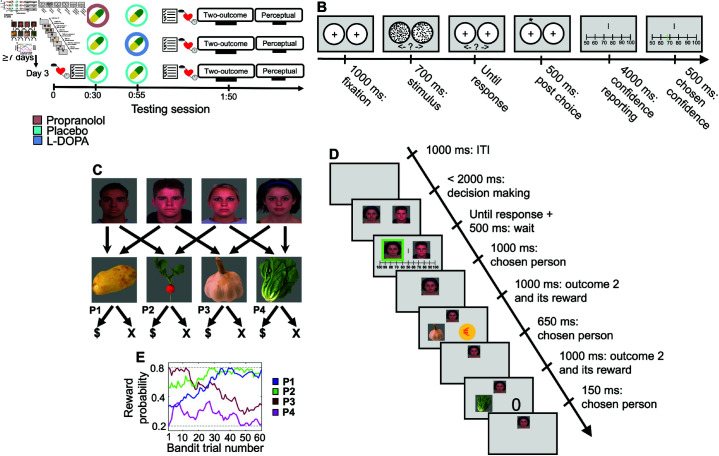
Experimental design and cognitive task. A) The experiment took place over three days; sessions were separated by at least one week and at most three weeks. The figure illustrates the sequence of tests of blood pressure, affective and meta-cognitive scores and the two cognitive tasks: the two-outcome confidence task and the perceptual decision-making task (separated by a 10 minutes rest period). 25 mins elapsed between the first and second administration of capsules, and 55 mins between the second administration of capsules and the start of the cognitive task. The drug and placebo administration were organized so that the procedure could be double-blind. B) The task involved a perceptual decision-making challenge. Participants were given the task of determining which of the two circles contained a greater density of dots. The densities were adjusted using a staircase procedure to ensure an appropriate performance level. Following their selection in each trial, participants were asked to express their confidence regarding their decision. C) Participants were introduced to four people and learned which unique combination of two vegetables each person grew. Each vegetable was grown by two different people. When offered for sale in a “village market”, vegetables were sold probabilistically according to their demand, providing one ‘bonus’ point of value. D) On each trial participants were asked to choose, within 2 sec, one of a pair of randomly-offered people. Next, participants rated their confidence (continuous scale between 50 and 100) that they had chosen the more rewarding person of the pair. At this point, participants discovered, for each vegetable (in random order), whether or not it sold. In the current example, the garlic but not the lettuce was sold, indicated by the presence or absence of gold coins. E) Across trials (one 60-trial block is illustrated), the market demand for the four vegetables drifted according to independent Gaussian random walks, with reflecting boundaries at 0.2 and 0.8.

  **Experimental design.** Out of the initial 35 subjects who participated in this study, 30 successfully completed the task (Table A in S1). The study was conducted using a within-subject design and spanned across three days. The administration of L-DOPA followed the guidelines outlined in Hauser et al. [23], and propranolol was administered based on the procedures detailed in Hauser et al. [22]. The assessment battery included a wide range of measurements, such as blood pressure readings (were analyzed in Box A of the S1), affective and meta-cognitive scores, and performance on two cognitive tasks: two-outcome confidence task and perceptual decision-making task. Note that the administration of both drugs and placebos was meticulously organized to ensure a double-blind procedure (Fig 1A).

Like Hauser et al. [22], we did not see any influence of our three drug conditions on mood ratings through the PANAS questionnaire [39] (elaborated on in Table B of the S1), (propranolol vs. placebo: b  = 0 . 433, t ( 326 ) = 0 . 574, p = 0 . 566, 95*%CI* = [ - 1 . 047 , 1 . 914 ] ; L-DOPA vs. placebo: b  = 0 . 716, t ( 326 ) = 0 . 949, p = 0 . 343, 95*%CI* = [ - 0 . 764 , 2 . 197 ] ; time: b  = 0 . 85, t ( 326 ) = 1 . 379, p = 0 . 169, 95*%CI* = [ - 0 . 357 , 2 . 057 ] ). Also the results from the MCQ-30 [40] questionnaire did not differ significantly between three drug conditions (propranolol vs. placebo: b  = - 0 . 062, t ( 58 ) = - 0 . 733, p = 0 . 466, 95*%CI* = [ - 0 . 234 , 0 . 108 ] ; L-DOPA vs. placebo: b  = - 0 . 048, t ( 58 ) = - 0 . 567, p = 0 . 573, 95*%CI* = [ - 0 . 219 , 0 . 123 ] ), confirming no significant difference in meta-cognitive ability before influence of drugs. In below, we would first report the results from the Perceptual task and then the two-outcome task.

### 2.1. Meta-cognitive ability in perceptual decision-making

  **Perceptual task.** The experiment involved a perceptual decision-making task with confidence assessment sourced from Fleming et al. [36]. Participants were asked to determine which of two large circles had a higher density of dots. An online control mechanism adjusted task difficulty to maintain consistent performance levels. The training phase familiarized participants with the task and established personalized difficulty levels. The main task consisted of eight blocks of trials where participants made choices using a computer mouse. They reported their confidence levels using a slider. An interval separated each consecutive trial (Fig 1B).

**Fig 2:**
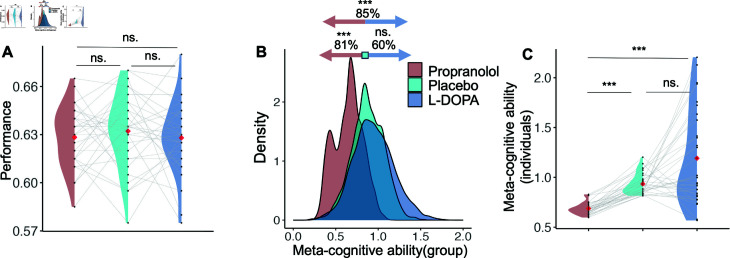
Influence of drug conditions in the Perceptual task. A) The performance, represented by black dots connected to each other by gray lines, is shown for each subject in three conditions. The average performance in each drug condition is indicated by red dots. The distribution of performance in each condition is color-coded as ’red’ for propranolol, ’green’ for placebo, and ’blue’ for L-DOPA conditions. Performance was not significantly different between three drug conditions. B) We assessed the group-level Meta-cognitive efficiency in each drug condition using M-ratio estimates obtained through Bayesian fitting, called H-meta. We observed that 81*%* of the group-level estimates were lower in the propranolol condition compared to placebo, suggesting reduced meta-cognitive ability under propranolol’s influence. This difference is illustrated by a red arrow pointing from the green square to the left (with the 81*%* mentioned above). Additionally, 60*%* of the samples in the L-DOPA condition were higher than those in the placebo condition, representing no influence of L-DOPA’s condition relative to placebo. The blue arrow pointing from the green square to the right indicates the direction of 60*%* of samples. Moreover, 85*%* of the samples in the L-DOPA condition were higher than those in the propranolol condition, depicted by a two-directional arrow with red on the left and blue on the right. C) We displayed individual-level estimates of meta-cognitive efficiency using black dots connected by gray lines in three drug conditions. The group-level averages of these estimates, along with their distributions, are shown as red dots and color-coded distributions for each drug condition. The comparison of meta-cognitive efficiency across drug conditions aligns with the group-level results.

There was no notable variation in decision-making performance between the propranolol and placebo conditions (b  = - 0 . 003, t ( 58 ) = - 0 . 778, p = 0 . 44, 95*%CI* = [ - 0 . 013 , 0 . 006 ] )or between the L-DOPA and placebo conditions (b  = - 0 . 004, t ( 58 ) = - 0 . 846, p = 0 . 401, 95*%CI* = [ - 0 . 014 , 0 . 006 ] ) (Fig 2A). Similarly, there was no effect on overall reported confidence, as shown by the statistical analysis, between propranolol and placebo (b  = - 0 . 801, t ( 58 ) = - 1 . 064, p = 0 . 292, 95*%CI* = [ - 2 . 31 , 0 . 71 ] )or between L-DOPA and placebo (b  = - 0 . 352, t ( 58 ) = - 0 . 468, p = 0 . 642, 95*%CI* = [ - 1 . 86 , 1 . 16 ] ).

We quantified meta-cognition in our three drug conditions using the M-ratio [4]. This measure represents the proportion of metacognitive sensitivity, meta-*d′*, to choice sensitivity, *d′*. The *d′* was estimated by fitting choice data to a model based on signal detection theory(SDT) and meta-*d′* by fitting both choice and confidence data to the same model. In estimating the M-ratio to enhance statistical power, incorporate uncertainty in group-level parameter estimates, and avoid edge correction confounds, we employed a hierarchical, Markov chain Monte-Carlo-based Bayesian estimation approach, called Hmeta (the method and package were from Fleming [41]) to fit the SDT model to data. This approach allows for estimating the M-Ratio across both individuals and conditions. Because there were the same subjects in our three conditions, we used a version of Hmeta in which each subject’s meta-cognitive efficiencies in the three conditions are specified as draws from a multivariate Gaussian distribution, M-ratio in each condition was in the role of a dimension of the Gaussian distribution. To match the method to our within-subject study design, the correlation between conditions was allowed [41] and variance for each dimension was the variance across subjects in the associated condition of experiment.

The posterior distribution of meta-cognitive efficiency in the placebo condition overlapped with the normative estimate of 1, as expected [41] (Fig 2B). At the group level, 81*%* of samples were lower under propranolol than placebo; 60*%* of samples were higher under L-DOPA condition than placebo; and 85*%* of samples were lower under propranolol than L-DOPA. We also randomly picked 30 samples from each distribution of M-ratio in the three drug conditions and compared them using the Wilcoxon rank-sum test. We repeated the described comparison 1,000 times; 99 . 6*%* of p-values were lower than 0.05 (mean  = 0 . 001, sd  = 0 . 005), confirming the decrease in metacognitive ability under the influence of propranolol (Fig E in S1, plots A and B). Only 55 . 5*%* of p-values were lower than 0.05 (mean  = 0 . 1, sd  = 0 . 144), rejecting the enhancement of metacognitive ability in L-DOPA relative to the placebo condition (Fig E in S1, plots C and D). In addition, 100*%* of p-values were lower than 0.05 (mean $=1.5e^{-5}$, sd $=1.32e^{-04}$), confirming lower metacognitive ability in the propranolol condition relative to L-DOPA (Fig E in S1, plots E and F).

We also used the average of M-ratio samples for each participant as an individual estimate of the M-ratio. The estimated meta-cognitive efficiency was lower for all subjects in the propranolol condition, whereas it was higher for 18 out of 30 individuals in the L-DOPA condition compared to the placebo condition. Additionally, the same estimate of meta-cognitive ability was lower in the propranolol condition compared to the L-DOPA condition for 26 out of 30 subjects. Across all subjects, the linear mixed effect model revealed decrease of M-ratio in propranolol condition relative to placebo one (b  = - 0 . 246, t ( 58 ) = - 3 . 425, p = 0 . 001, 95*%CI* = [ - 0 . 388 , - 0 . 105 ] ) and increase of M-ratio in Dopamine condition relative to placebo one (b  = 0 . 257, t ( 58 ) = 3 . 577, p$=7.1e^{-4}$, 95*%CI* = [ - 0 . 388 , - 0 . 105 ]) L-DOPA (Fig 2C). The posthoc non-parametric test showed lower M-ratio in propranolol relative to placebo condition (W  = 7, p$=7.61e^{-16}$). However, there was no significant difference between the L-DOPA and placebo conditions (W  = 541, *p* = 0 . 182); furthermore, propranolol decreased the M-ratio also relative to the L-DOPA condition (W  = 82, *p* = 2 . 419*e* - 9).

The group-level distribution of correlation between M-ratio of each two drug conditions was symmetric around zero (propranolol and placebo; mean = 0 . 091, sd  = 0 . 479, L-DOPA and placebo; mean  = 0 . 013, sd  = 0 . 479, propranolol and L-DOPA; mean  = 0 . 047, sd  = 0 . 474), so there was not considerable correlation between meta-cognitive ability in drug conditions and placebo condition. In other words, the drug did not influence subjects’ behavior monotonically.

### 2.2. Modulating control and meta-control in MF and MB learning

  **Two-outcome task.** Participants were presented with four people, each associated with specific vegetables. On each trial, they had to choose between two of the people to receive rewards from the associated vegetables, with rewards changing probabilistically. Participants first learned the associations between people and vegetables, going through learning and quiz phases. After learning, they had practice trials to ensure comprehension, followed by five blocks of trials. Each trial involved a choice between two people who shared a common vegetable. After making a choice, participants rated their confidence using a continuous scale. They then discovered the rewards for their chosen vegetables. The reward probabilities for the vegetables changed across trials. There were breaks between blocks, and participants were informed that reward probabilities were reset after each block (Fig 1C).

#### MF and MB contributions to choice.

**Fig 3:**
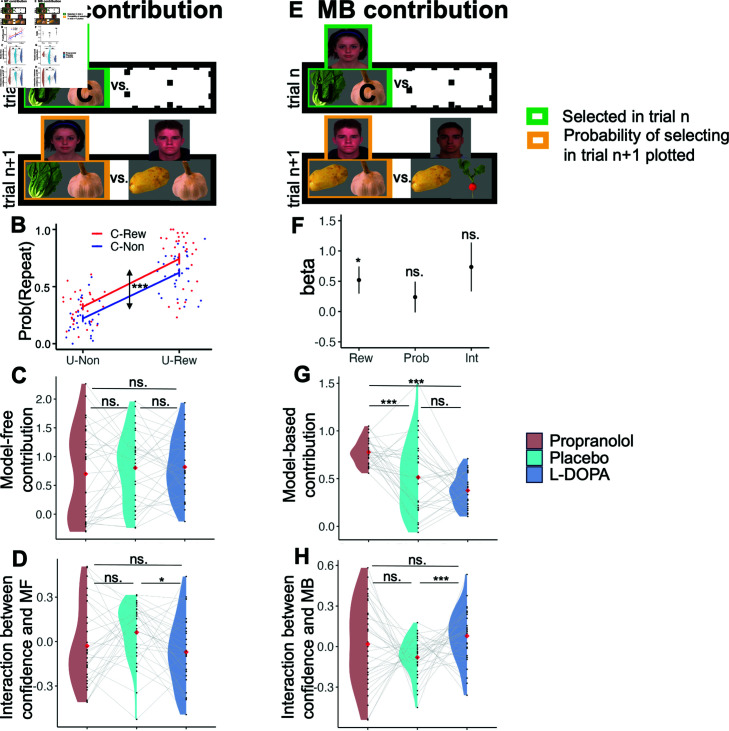
MF and MB contributions to choice and interaction with confidence. A) To assess the contribution of a MF system, we analysed the subset of trials that included on trial *n* + 1 the person chosen on trial *n*. For clarity, we represent people by their associated pair of vegetables; in the actual experiment, participants only saw images of the people. Places for people whose identity did not affect the analysis (and were marginalized over) are left empty. B) The empirical probability of repeating a choice as a function of the previous-trial Common (“C”), and Unique (“U”) outcomes. The main effect of the Common outcome highlights a MF contribution to bandit choices. C) The MF contribution was compared across our three drug conditions. There was no significant difference between drug conditions. D) We compared the three drug conditions regarding the relationship between low versus high confidence on one trial and the MF contribution to choice on the subsequent trial. While propranolol showed no significant effect, L-DOPA reduced this interaction.

We followed the analysis strategy of Moran et al. [27,42,43] to check that we could replicate the combined involvement of MF and MB reasoning in the placebo condition. Consider a trial n+1, which offers for choice the person chosen on trial n (e.g., the girl), against another person (e.g., the young man with brown hair; Fig 3A). The two offered people grow a “Common vegetable” (the garlic, denoted C) but the trial-n chosen person also grows a “Unique vegetable” (the lettuce, denoted U). We tested whether the probability of repeating a choice depended on the trial n Common-vegetable outcome, while controlling for the Unique-vegetable outcome. A positive Common reward effect is a signature of MF contribution to choices, since the MB system would note that the benefit of the Common-vegetable would favour both trial n+1 choices.

Using a logistic mixed effects model, in which we regressed the probability of repeating the trial-n choice on Common and Unique trial-n outcomes, we found a main effect for the Common outcome, $C_{MF}$, (b = $0.83,t(116)=5.519,p=2.1007e^{-07},95\%$CI =  [ 0 . 532 , 1 . 128 ] )(Box B in S1). This effect was qualified weakly by an interaction between Common and Unique outcomes, $C_{MF}:U_{MF}$, (b = 0 . 398 , *t* ( 116 ) = 0 . 398 , *p* = 0 . 044 , 95*%CI* = [ 0 . 011 , 0 . 785 ] ). Simple effects analysis showed that the effect of reward was significantly positive both when the unique outcome was rewarded (b$=1.022,t(58)=4.799,p=1.155e^{-5},95\%CI=[0.595,1.449]$), and unrewarded (b$=0.627,t(58)=4.834,p=1.018e^{-5},95\%CI=[0.368,0.887]$). Thus, the MF contribution in Moran et al. [27,42,43] was replicated in our placebo condition (Fig 3B and Table 1).

Turning next to the MB contribution, consider a trial n+1 which excludes the person chosen on trial n (e.g., the girl; Fig 3E) from the choice set. In this case, the trial *n* chosen person shares a Common vegetable (onion) with only one of the trial *n* + 1 offered people (e.g., the young man with brown hair). It has been argued that a positive Common reward effect on choice generalization (after controlling for the generating reward probability of this common outcome, explained at Moran et al. [27]) constitutes a signature of MB contributions to choices [27]. A logistic mixed effects model showed (Eqs 1 and 2 in S1) a positive main effect for the Common-outcome on choice-generalization, supporting a MB contribution to bandit choices, $C_{MB}$, (b  = 0 . 519 , *t* ( 4340 ) = 2 . 327, *p* = 0 . 02, 95*%CI* = [ 0 . 082 , 0 . 958 ] ). We found no significant effect of the reward probability of the common vegetable, $Prob_{MB}$, (b  = 0 . 239, *t* ( 4340 ) = 0 . 942, *p* = 0 . 346, 95*%CI* = [ - 0 . 258 , 0 . 736 ] ), nor any interaction between the common trial-n outcome and the reward probability of the common vegetable, $C_{MB}:Prob_{MB}$, (b  = 0 . 736, *t* ( 4340 ) = 1 . 829, *p* = 0 . 068, 95*%CI* = [ - 0 . 053 , 1 . 525 ] ). In other words, the generalization behavior was influenced by the outcome of the common vegetable in trial n, but not by its reward history. Thus, the MB contribution in Moran et al. [27,42,43] was replicated in the placebo condition (Fig 3F and Table 2).

We also checked the significance of MB and MF contributions in propranolol and L-DOPA conditions. The MF and MB contributions in propranolol condition were significant (MF contribution ($C_{MF}$); b  = 0 . 726 , *t* ( 116 ) = 4 . 025, $p=1e^{-4}$, 95*%*CI = [ 0 . 352 , 1 . 2 ] , MB contribution ($C_{MB}$) ; b  = 0 . 776 , *t* ( 4290 ) = 3 . 59, $p=3e^{-4}$, 95*%*CI = [ 0 . 369 , 1 . 083 ] ). The MF contribution was significant in the L-DOPA condition while the MB was not significant (MF contribution ($C_{MF}$); b  = 0 . 849 , *t* ( 116 ) = 5 . 904, $p=3.599e^{-8}$, 95*%*CI = [ 0 . 564 , 1 . 134 ] , MB contribution ($C_{MB}$); b  = 0 . 377 , *t* ( 4305 ) = 0 . 209, *p* = 0 . 07, 95*%*CI = [ - 0 . 033 , 0 . 788 ] ) (Tables 1 and 2).

**Table 1:** MF contributions. The coefficients come from logistic mixed-effects regressions. $C_{MF}$ and $U_{MF}$ represent the rewards from common and unique outcomes, respectively. Their interaction is denoted by $C_{MF}:U_{MF}$. The gray numbers indicate p-values.

	*C_MF_*	*U_MF_*	*C_MF_* : *U_MF_*
**Propranolol**	$\overset{***}{0.726}\left(<0.001\right)$	$\overset{***}{1.93}\left(<0.001\right)$	$\underset{\left(0.216\right)}{0.243}$
**placebo**	$\overset{***}{0.83}\left(<0.001\right)$	$\overset{***}{2.203}\left(<0.001\right)$	$\overset{*}{0.398}\left(0.044\right)$
**L-DOPA**	$\overset{***}{0.849}\left(<0.001\right)$	$\overset{***}{2.256}\left(<0.001\right)$	$\underset{\left(0.483\right)}{0.164}$

**Table 2:** MB contributions. The coefficients are from logistic mixed-effects regressions. $C_{MB}$ represents the rewards from common outcomes, and $Prop_{MB}$ represents the history of these rewards. Their interaction is shown as $C_{MB}:Prop_{MB}$. The gray numbers indicate p-values.

	*C_MB_*	*Prob_MB_*	*C_MB_* : *Prob_MB_*
**Propranolol**	$\overset{***}{0.776}\left(<0.001\right)$	* 0.608 (0.032)	$\underset{\left(0.581\right)}{0.27}$
**placebo**	$\overset{*}{0.519}\left(0.02\right)$	$\underset{\left(0.346\right)}{0.239}$	$\underset{\left(0.068\right)}{0.736}$
**L-DOPA**	$\underset{\left(0.071\right)}{0.377}$	$\underset{\left(0.412\right)}{0.286}$	$\overset{*}{1.06}\left(0.04\right)$

To compare these effects in our three drug conditions, we assessed the total (fixed plus random; Methods and also Box C in S1) effect from the described logistic mixed effect regression for MF and MB contribution for each of three drug conditions.

The MF contribution did not differ significantly between the propranolol and placebo conditions (b  = - 0 . 106, t ( 58 ) = - 835, p = 0 . 407, 95*%CI* = [ - 0 . 36 , 0 . 148 ] ), nor between the L-DOPA and placebo conditions (b  = - 0 . 106, t ( 58 ) = - 835, p = 0 . 407, 95*%CI* = [ - 0 . 24 , 0 . 27 ] )(Fig 3C). The propranolol condition resulted in a significant decrease in MB contribution compared to the placebo condition (b  = 0 . 263, t ( 58 ) = 4 . 058, p$=1.08e^{-4}$, 95*%CI* = [ 0 . 134 , 0 . 392 ] ). Conversely, the L-DOPA condition led to a significant increase in MB contribution relative to the placebo condition (b  = - 0 . 138, t ( 58 ) = - 2 . 136, p = 0 . 035, 95*%CI* = [ - 0 . 267 , - 0 . 009 ] ) (Fig 3G). The posthoc non-parametric test showed higher MB contribution in propranolol relative to placebo condition (W  = 670, $p=9.2e^{-4}$) and no significant difference between L-DOPA and placebo ones (W  = 359, *p* = 0 . 182); furthermore, propranolol increased the MB contribution also relative to the L-DOPA condition (W  = 878, $p=7.62e^{-14}$).

Furthermore, we compared the behavior of subjects in three drug conditions via the hybrid reinforcement model [27,42,43](Box D in S1). The likelihood from fitting the hybrid model to empirical data was not significantly different between the propranolol and placebo conditions (*b* = - 0 . 043, *t* ( 58 ) = - 0 . 014, *p* = 0 . 989, 95*%CI* = [- 6 . 265 , 6 . 177 ] ), similarly between L-DOPA and placebo conditions (*b* = 0 . 448, *t* ( 58 ) = 0 . 144, *p* = 0 . 886, 95*%CI* = [ - 5 . 772 , 6 . 669 ] ). Then, we compared the parameters of the model which represented MF and MB behavior. There was no significant difference in MF weights between the propranolol and placebo conditions (*b* = - 0 . 64, *t* ( 58 ) = - 1 . 058, *p* = 0 . 295, 95*%CI* = [ - 1 . 850 , 0 . 571 ] ) or between the L-DOPA and placebo conditions (*b* = - 0 . 766, *t* ( 58 ) = - 1 . 267, *p* = 0 . 21, 95*%CI* = [ - 1 . 977 , 0 . 445 ] ) (Fig 2A). Likewise, there was no observed effect on MB weights, as indicated by the statistical analysis, between propranolol and placebo (*b* = - 0 . 145, *t* ( 58 ) = - 0 . 613, *p* = 0 . 542, 95*%CI* = [ - 0 . 621 , 0 . 33 ] ) or between L-DOPA and placebo (*b* = - 0 . 174, *t* ( 58 ) = - 0 . 732, *p* = 0 . 467, 95*%CI* = [ - 0 . 649 , 0 . 301 ] )(Fig A in S1).

To confirm the discrepancy between the modeling results and the results from regression analysis, we ran the regression analysis on the simulated behavior of the RL model, based on empirical parameters in each of the three conditions of task. Then, we used average of MB behavior from regression analysis across all simulations as an estimation of synthetic MB behavior for each subject in each condition. The estimated synthetic MB behavior was not significantly different between our three drug conditions (propranolol vs. placebo: b  = - 0 . 062, t ( 58 ) = - 0 . 733, p = 0 . 466, 95*%* CI  = [ - 0 . 234 , 0 . 108 ]  ; L-DOPA vs. placebo: b  = - 0 . 048, t ( 58 ) = - 0 . 567, p = 0 . 573, 95*%* CI  = [ - 0 . 219 , 0 . 123 ] ) (Fig C in S1). Thus, the simulated behavior based on the hybrid model did not reflect the variations observed in the empirical data when viewed through regression analysis.

#### Meta-cognition and meta-control in MF and MB learning.

**Meta-cognition.** We then examined the influence of drug conditions on second-order decision-making in the two-outcome task. The average confidence of participants was unaffected by the propranolol condition (b  = - 0 . 804, t ( 58 ) = - 0 . 728, p = 0 . 47, 95*%* CI  = [ - 3 . 02 , 1 . 41 ] ) and likewise by the L-DOPA condition (b  = - 0 . 873, t ( 58 ) = - 0 . 79, p = 0 . 433, 95*%* CI  = [ - 3 . 09 , 1 . 34 ] ). We also assessed meta-cognitive ability in the two-outcome task using the Quadratic Scoring Rule (QSR) [3]. A mixed-effects linear analysis indicated that the propranolol condition reduced meta-cognitive ability (b  = - 0 . 013, t ( 58 ) = - 2 . 385, p = 0 . 02, 95*%* CI  = [ - 0 . 025 , - 0 . 002 ] ), while the L-DOPA condition showed no significant effect (b  = - 0 . 005, t ( 58 ) = - 1 . 008, p = 0 . 318, 95*%* CI  = [ - 0 . 017 , 0 . 006 ] ). Although the reduction in meta-cognitive ability under propranolol in the two-step task aligned with the observed effect in the perceptual task, posthoc analysis did not confirm a significant effect of drug conditions on meta-cognitive ability in the two-outcome task (Propranolol vs. placebo: W = 319, p = 0.053; L-DOPA vs. placebo: W = 393, p = 0.406; Propranolol vs. L-DOPA: W = 357, p = 0.173).

**Meta-control.** In order to investigate the interaction between confidence and MF/MB behavior, as well as the effects of different drugs, we examined the influence of the MB system using trial transitions of the type depicted in Fig 3A and 3E, testing whether the effect of a Common reward at trial *n*, $C_{MF,n}$ and $C_{MB,n}$ respectively, on choice repetition (for MF, Eq 1) and generalization (for MB, Eq 2) depends on whether the expressed trial *n* confidence was high or low, $Conf_{hl,n}$ (we conducted separate analyses for MF and MB components). We used logistic mixed effects model to assess the interaction of Common reward and previous-trial confidence (high or low, higher or lower than median for each subject, coded by 0.5 or –0.5) on choice repetition and generalization for each drug condition.

Then, we compared the interaction of confidence with MF and MB behavior across all subjects between three drug conditions through linear mixed effect model. Although there was not a significant influence of propranolol condition on the interaction between confidence and MF behavior in the total effect from the mixed effect regression (b  = - 0 . 091, t ( 58 ) = - 1 . 624, p = 0 . 11, 95*%CI* = [ - 0 . 204 , 0 . 021 ] ), there was a decrease in this interaction in the L-DOPA condition relative to the placebo condition (b  = - 0 . 133, t ( 58 ) = - 2 . 356, p = 0 . 022, 95*%CI* = [ - 0 . 246 , - 0 . 020 ] )(Fig 3D). The post-hoc analysis also revealed that the propranolol condition had no significant influence on the interaction (W  = 332, *p* = 0 . 08) and that the L-DOPA condition resulted in a decrease in the interaction (W  = 282, *p* = 0 . 012). Furthermore, the interaction did not differ between propranolol and L-DOPA conditions (W  = 472, *p* = 0 . 752). Regarding the influence of drug conditions on the interaction between confidence and MB behavior, the propranolol condition did not have a significant effect on the interaction (b  = 0 . 097, t ( 58 ) = - 1 . 64, p = 0 . 104, 95*%CI* = [ - 0 . 021 , 0 . 216 ] ), whereas the L-DOPA condition increased the interaction (b  = 0 . 158, t ( 58 ) = 2 . 66, p = 0 . 009, 95*%CI* = [ 0 . 040 , 0 . 276 ] ) (Fig 3H). The Post-hoc analyses indicated that the propranolol condition did not significantly influence the interaction (W  = 538, *p* = 0 . 197). In contrast, the L-DOPA condition resulted in a significant increase in the interaction (W  = 677, $p=6.1e^{-4}$). Furthermore, there was no significant difference in the interaction between the propranolol and L-DOPA conditions (W  = 400, *p* = 0 . 467).


$$\begin{array}{c} Repeat_{n+1}\sim C_{MF,n}+Conf_{hl,n}+(C_{MF,n}:Conf_{hl,n}|participant) \end{array}$$ (1)

$$\begin{array}{c} Generalization_{n+1}\sim C_{MB,n}+Conf_{hl,n}+(C_{MB,n}:Conf_{hl,n}|participant) \end{array}$$ (2)

**Fig 4:**
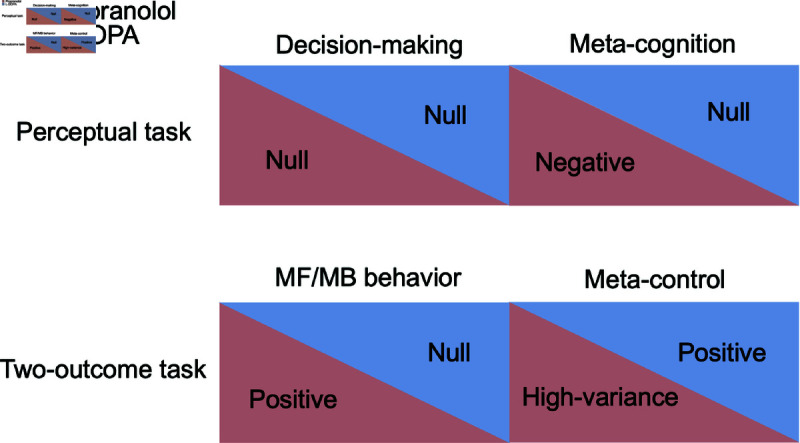
Overview of results. Propranolol and L-DOPA did not influence decision-making in the perceptual task; however, metacognitive ability was decreased in the propranolol condition and was not affected in the L-DOPA condition. MB behavior was boosted under propranolol, but was not significantly influenced by L-DOPA. Although the meta-control in propranolol condition was highly variable, it was significantly higher in L-DOPA relative to placebo condition.

## 3. Discussion

We examined the consequences for meta-cognition and meta-control of pharmacologically manipulating two key neuromodulators, norepinephrine (via propranolol) and dopamine (via L-DOPA). Following Hauser et al. [22], we investigated meta-cognitive confidence in a perceptual decision-making task. We found that neither of the drugs had a significant impact on perceptual performance compared to the placebo condition. Our results did suggest that propranolol but not L-DOPA, influenced meta-cognitive efficiency compared to placebo, respectively. Also meta-cognitive ability was significantly lower under propranolol relative to L-DOPA.

We investigated meta-control in a novel version of a two-outcome task [27] in which we asked subjects for their confidence in their choices on a trial-by-trial basis, and so could correlate their reports with their ongoing use of MB and MF methods of choice. In this task, propranolol, but not L-DOPA, resulted in a greater influence of the MB system on behaviour relative to placebo and also L-DOPA condition. Neither drug influenced the MF contribution to choice. Nevertheless, L-DOPA, decreased/increased the interaction between confidence on a trial and MF/MB behavior on the next trial compared to placebo, but not relative to the propranolol condition. In the latter case, the size of the interaction was highly variable. In summary, our results across two rather different tasks pointed toward disrupted meta-cognition and meta-control under propranolol and the maintenance, or even mild improvement, in individuals under L-DOPA (Fig 4).

**Propranolol:** Reflecting the very broad impact of the noradrenergic system, this drug is indicated for various anxiety and stress-related conditions, including stage fright, post-traumatic stress disorder (PTSD), arachnophobia, and autism spectrum disorder [e.g., 44–48].

Our finding that propranolol did not affect first-order performance in the perceptual decision-making task is consistent with the results of Hauser et al. [22]. However, whereas they showed that the drug had a positive effect on meta-cognitive sensitivity (measured by the area under the type-2 receiver operator characteristic curve), the Hmeta results did not show any significant influence of meta-cognitive efficiency. Hauser et al. [22] suggested two potential reasons for the improvement they found based on the normal function of NE on increasing neural gain [14] or on resetting aspects of neural processing [16]. In the former case, gain increases might limit the detail available to meta-cognitive processing; in the latter, a reset following a response that error-monitoring suggests might be erroneous could have impaired meta-cognition. Thus, these two potential direction of influence of propranolol on meta-cognitive efficiency might be the sources of the decrease we observed.

We can only speculate about the source of the difference between Hauser et al. [22]’s and our results. First, the observation that propranolol boosted the impact of the more cognitively-demanding MB system in the two-outcome task suggests that there is no general reduction in the capacity for more sophisticated aspects of cognition. Looking elsewhere, one general possibility arises from the notorious inverted U-shaped curves of action of neuromodulators [49]. For instance, it’s possible that the subjects’ arousal levels decreased by the time they reached the perceptual task at the end of the experiment, leading to a diminished effect of propranolol on their meta-cognitive abilities, which involve more complex cognitive processes.

We are not aware of a previous investigation of the influence of propranolol on MF and MB value-based decision-making, let alone on the relative influence of the two systems in choice. There is evidence from a different task that stress of various sorts reduces MB influences over control, perhaps by interfering with working-memory [28,50]. Thus, propranolol’s anxiolytic action would be consistent with a boost to MB behavior perhaps also consistent with decreased heart rate in propranolol relative to other conditions (Fig D in S1, plot B). However, it was notable that the effect we observed was only evident in the one-trial-back logistic regression analysis, and not in the multi-trial analysis that utilized a dual reinforcement learning approach. This suggests that Propranolol might influence an additional aspect of behavior not yet fully explored in the two-outcome task. This could involve a direct working-memory contribution to model-based control, similar to its effects in model-free control as indicated by prior research [51]. This uncharted area of influence merits further investigation to understand its role in two-outcome task fully.

We currently lack a suitable measure for meta-cognitive efficiency in value-based decision-making problems such as our two-outcome task that might parallel suggestions such as the M-ratio [4,41]. This is because that measure is biased when the underlying difficulty of the problem is not constant [52], something that is inevitable in the two-outcome task as the qualities of the outcomes fluctuate. However, it was unexpected that propranolol did not affect meta-control in the task and induced a high variability. Of course, even when there is a more straightforward method of assessment, choice- and confidence-related cognitive abilities do not always perfectly covary [41,53].

**L-DOPA:** Manipulating dopamine has been found to affect many aspects of cognitive function including reinforcement learning, working memory, self-monitoring, planning and reasoning [12,24,34,54–58].

In terms of the perceptual task, the lack of effect of boosting dopamine on performance was largely to be expected [22,24]. The null effect on meta-cognitive efficiency in our results was in line with no influence of L-DOPA on meta-cognitive ability found by Hauser et al. [22], although different drugs (L-DOPA versus amisulpride) were administrated. The impairment in meta-memory found by Clos et al. [24] and the difference with our result could be related to differences of meta-cognitive neural system across domains [36,59].

The lack of effect of L-DOPA on the magnitude of MB influence in the two-outcome task was less expected, since boosting dopamine or its effects have previously been shown to boost this influence in the two-step task [23], albeit also with no effect on aspects of MF control. However, our finding is consistent with evidence from a slightly more complicated version of the two-outcome task (including uncertainty trials which a ghost made decision instead of participant) in which there was also no significant effect of L-DOPA on the measure of MB control that we also employed [21]. In fact, our original expectation in the two-step task was that dopamine’s influence over MF processing [60] might have made it more dominant, rather than less [23]. Thus, perhaps, in the two-outcome task, these two excess influences canceled out [61]. It is also questioned whether the MF and MB behaviors were dissociable in this task. As Feher da Silva and Hare [62,63] studied, a lack of understanding about the structure of the task could result in incorrectly taking the MF and MB behaviors for one another. The authors showed that with a clear and sensible storyline for learning the task structure, this problem could be avoided. In our study, a clear and relatable storyline was used to help participants understand the task structure, in addition to training until participants could pass quizzes. Thus, the MF and MB behaviors were dissociable in our study.

We did find evidence for an effect of L-DOPA on the interaction between confidence and our one-trial measure of MF/MB control. Here, low or high confidence on trial *n* respectively diminished or boosted the ’common-reward’ MB effect on trial *n* + 1 to a significantly weaker/greater degree under the drug. One might see this refined effect in relation to the modulation by L-DOPA that Deserno et al. [21] observed. That study included trials in which a notional ’ghost’ chose one of a pair of options. Only the MB system could infer which option had actually been picked (based on the outcomes that were observed); the issue is then whether this inference is propagated to the MF system so that it can update the value of that vehicle. Deserno et al. [21] showed that this inference or propagation was boosted under the influence of L-DOPA. In our task, it could be that a MB influence over MF control was similarly boosted – meaning that the choices that we reported as being consistent with MB influences might partly be implemented by an adjusted MF system (for instance by the sort of replay found in Eldar et al. [9] and analyzed by Antonov et al. [64], following Mattar et al. [65]). If the judgement of confidence was particularly determined by the MB system, then it might decline to update the MF system when it was unsure. It would be interesting to meld the two designs – asking for subjects’ confidence ratings in the version of the task run by Deserno et al. [21].

According to Morales et al. and Ais et al. [38,66], evidence exists for both domain specificity and domain generality in aspects of meta-cognition. We administered two tasks in two domains, perceptual and MF/MB learning, and observed the influence of drug conditions on meta-control in the later domain, but not in the former one. However, we did not find a correlation between meta-cognition in the perceptual task (individual level analysis; Fig 2C) and meta-control in the two-outcome task, which is line with discrepancy of results across two domains. It is also noticeable, that lack of a suitable measure for meta-cognitive efficiency in the two-outcome task [because difficulty is not constant;^52^] prevented us from comparing this between the two domains.

Furthermore, our results revealed an unusual pattern of variability among the conditions. In the perceptual task, there was relatively equal variance of performance across the conditions, but a much larger variance of meta-cognitive efficiency was observed for L-DOPA in individual posteriors. In the two-outcome task, while the variance was similar across conditions for MF contribution, it was significantly larger for MB contribution in the placebo condition compared to the other conditions. Moreover, the variance was roughly similar for the interaction of confidence and MF contribution across drug conditions, whereas for the similar interaction between confidence and MB contribution, the variance was larger in the propranolol condition compared to the other conditions. We could not identify any clear reasons for these variance differences, and they may have influenced the statistical power.

In conclusion, we offer our study as a contribution to the understanding of the influence of neuromodulators on more global aspects of cognition. We found that propranolol and L-DOPA had subtle effects on learning and confidence, via a novel variant of a task exploring model-based and model-free contributions to choice, to meta-control. Understanding the fuller scope of these effects in a wider range of tasks, and assessing the effect of other neuromodulators that can also influence aspects of meta-processing, is a compelling task for the future.

## 4. Materials and methods

### 4.1. Ethics statement

This study was approved by the Regional Research Ethics Committee of the Faculty of Psychology and Education at the University of Tehran, Iran (protocol IR.UT.PSYEDU.REC.1399.022). All participants provided written informed consent prior to participation.

### 4.2. Experimental paradigm

Thirty-five subjects (17 female, aged 18-30) participated in this study, with thirty of them completing the task (additional details are provided in Table A of the S1). The subjects performed the task three times, each separated by at least a week and at most three weeks. The sample size was determined based on the effect size reported by Hauser et al. [22]. To achieve a significance level (*α*) of 0.05 and a power (*β*) of 0.95, sample size estimation was performed using G*Power 3.1. Experiments were run at similar times in the mornings, taking a total of around 3 hours and 40-50 mins per session. Participants were recruited through advertisements. The payment was according to their performance plus a baseline amount for participating in our task.

We used L-DOPA (Levodopa-B, 125 mg; 100 mg Levodopa, 25 mg Benserazid, catalyzes the conversion of levodopa into dopamine; Neurostar) according to Wunderlich et al. [23] and propranolol (HCL, to improve water solubility, 40 mg) based on Hauser et al. [22].

**Drug administration.** Drugs were presented in the form of capsules, and were taken with mineral water. Subjects were invited to eat a sweet snack if they felt hungry; they were also allowed to eat a salty snack if they subjectively felt low blood pressure after taking the drug. Drug administration was double blind ; two capsules were taken 80 and 55 mins before performing the task because of the different speeds with which the active compounds take effect [22,23] (Fig 1A). The order of the three experimental conditions was randomized for each subject using the ’Sample’ function in R software.

**Health measurements.** After reading and signing the consent form, subjects completed health questionnaires before taking the drugs, the questions were according to Guitart-Masip et al. [67] and also protocols of university of Tehran. These included questions about: a) normal eating habits and what had been consumed on the day of the experiment; b) normal use of cigarettes, different types of tobacco, alcoholic drinks and any consumption of these on the day of the experiment; c) any previous surgeries; d) any unusual headache, sleep disorder, weakness or low blood pressure, mental crisis, depression or signs of stress in the past, specially in the previous month; e) handedness (defined by use for writing). The subjects’ weight and height were recorded. A doctor measured the blood pressure and heart rate before drug administration. Subjects were excluded if their blood pressure was lower than 60/120, their heart rate was lower than 60 beats per minute. Three subjects were excluded because of low blood pressure, and two because of the drugs and some kind of tobacco that they used in previous weeks. Also, the doctor asked them, in addition to our questionnaire, if they suffered from a set of diseases, including asthma, arrhythmia disease, depression, diabetes, thyroid and various kinds of kidney, liver, heart diseases, which none of our subjects suffered from them. The doctor measured the subjects’ blood pressure and heart rate for the second time before doing the task to exclude anyone who was out of the normal (described above) range under influence of drug. No one was excluded in the final check up.

**Cognitive questionnaires.** Before taking the first capsule, the subjects completed the translated version of Positive and Negative Affect questionnaire (PANAS), which took around 10 minutes and collected information about feelings such as hostility, alertness, etc [39]. After using the first capsule, they completed a validated Persian translation (Yousefi et al. 2006) of the meta-cognition Questionnaire-30 (MCQ-30) which took around 15-25 minutes [40]. Finally, 20 mins before performing the task, the subjects repeated the PANAS questionnaire. The purpose of the repeat was to assess the influence of propranolol on mood [22], but we used this test on all three days to maintain the integrity of the double blind procedure.

**Drug administration.** The first author, S.E., utilized the ’sample’ function in R software to perform randomization and ordered capsules for 35 subjects. The second author, S.A., enrolled the participants and assigned them to the specified order, which had been prepared by S.E. and kept hidden from her. Recruitment took place in the year 2021.

### 4.3. Experimental tasks

The subjects performed the two-outcome task and, after a rest of 5-10 minutes and the opportunity to eat a sweet snack, the perceptual task. They did the tasks three times - once on each day. The two-outcome task, including training, took an average of 77.54 minutes, ranging from 55 to 118, with a standard deviation of 11.63. In addition, the combination of the break plus the perceptual task including the training averaged 24.74 minutes, spanning 10 to 82, with a standard deviation of 10.64. We first explain the Perceptual task because it is common in the literature for measuring meta-cognitive sensitivity.

**Perceptual task:** The experiment utilized a dot-density perceptual decision-making task with confidence assessment, sourced from the following GitHub repository: https://github.com/metacoglab/metadots [36]. Participants were tasked with determining which of two large circles contained a higher density of dots. To maintain consistent performance levels (65% accuracy) between subjects an online control mechanism adjusted the difficulty using a one-up, two-down staircase procedure. This ensured an adequate number of correct and error trials for assessing meta-cognitive efficiency. The training phase involved presenting example stimuli, with accompanying text indicating the dot count in each circle (e.g., 40 vs. 60). In a subsequent training phase, participants made judgments without providing confidence ratings. This phase familiarized them with the task and established a personalized difficulty level through the staircase procedure. The final phase comprised 10 practice trials emulating the main task, enabling participants to become accustomed to indicate their confidence levels. The main task consisted of eight blocks, each containing 25 trials. Each trial began with 1000 ms fixation on two empty circles, followed by a 700 ms presentation of the dots within the circles. Participants had unlimited response time and could select their choice by left or right-clicking on the mouse corresponding to the left or right circle, respectively. Subsequently, a star briefly appeared above the chosen circle for 500 ms. To report their confidence, participants had 4 s to adjust a vertical black line along a horizontal slider ranging from 50 to 100. Clicking on the line indicated their confidence level, with a left or right click corresponding to the left or right choice, respectively. A green-colored vertical black line was displayed for 500 ms to represent their selection. There was a 1000 ms interval between each consecutive trial (Fig 1B).

**Two-outcome task:** In this task, there are four ’people’ (represented by pictures), each of whom grows two out of four ’vegetables’ (also represented by pictures). On each trial, participants would be faced with a choice between two of the people; and would receive rewards from both the vegetables that their chosen person grows. The rewards were probabilistic, evolving according to a random walk.

Participants were first familiarised with four pictures of people, and learned which pair of vegetables each person grows. The pictures were taken from previous studies, vegetables from Kiani et al. and Kriegeskorte et al.[68,69] and faces from DeBruine and Jones [70]. Each vegetable was grown by two different people, and each person grew a unique pair of vegetables (Fig 1C). The mapping between people and vegetables was created at random for each participant. After each phase of learning, participants were quizzed about which vegetables each person grew and about which person they would choose to obtain a target vegetable. Participants iterated between learning and quiz phases until they achieved perfect quiz performance (100% accuracy and RT < 3000 ms for each question). This took a mean of 3.2 rounds (range 1–16, sd  = 2 . 553).

After learning, subjects played 8 practice trials, to verify that they understood the task. These practice trials were just like normal trials, except without a time limit on the choices. Subjects next played 5 blocks, each comprising 60 trials. On each trial, two of the four people were offered, and subjects had 2 seconds to choose between them (using a left or right mouse buttons). The people offered always grew one vegetable in common. This defined four possible people pairs, each of which was presented on 15 trials, in a random order.

Following each choice, participants rated their confidence in the correctness of their choice, on a continuous scale. The confidence scale was presented underneath, and equidistant from, the bandits (which remained on the screen). It started from 50 in the middle of the screen, finishing at 100 at both left and right ends. The numbers were seen on the line. A cursor appeared in the middle of the scale, and subjects were asked to move it with the mouse continuously and then click the same button that they used for their choice. In the training session, they experienced how to report their confidence. They saw that with wrong click, pressing left bottom while the choice was right and vice versa, nothing happens until they press the bottom congruent with their choice.

After rating their confidence, participants saw in turn whether each of the two vegetables that the chosen person grows provided a treasure (1 bonus point) or nothing (0 bonus point). The reward probabilities of the four vegetables evolved across trials according to four independent Gaussian random walks (with a standard deviation of 0.03 per trial) with reflecting boundaries at *p* = . 2 and *p* = . 8 (Fig 1E). After each block was completed participants had a forced break, minimum 1 and maximum 5 minutes. After the break, participants were informed that the reward probabilities for all the vegetables had been reset to new values, and therefore they should forget any previous impression about how rewarding different vegetables and people are, and form new impressions when the task resumes (Fig 1D).

In 69 out of the 90 sessions of our experiment, two random walks (as presented in Fig F of S1) were randomly associated with the drug conditions. The starting points were counterbalanced across these two random walks, and the probabilities of reward associated with vegetables did not remain consistently high or low for most of the trial. In the remaining 21 sessions, new random walks were randomly assigned to different sessions, with randomized starting points.

### 4.4. Data analysis

  **Perceptual task.** To assess meta-cognition in the perceptual task, we used the Hmeta package [41] for implementing a hierarchical Bayesian model. Analysis was conducted using JAGS version 4.3.1, with three Markov chains each generating 12 , 000 samples, which included 2 , 000 warm-up iterations per chain. A thinning rate of 1 was applied. Convergence was achieved with a tolerance of $1\times 10^{-5}$. The coda package was utilized for extracting the samples. The meta-cognitive efficiencies in the three conditions being assumed to be drawn from a multivariate Gaussian distribution, each variate associated with a task condition. We obtained estimates of meta-cognitive efficiency at both the group and individual levels. For the group level analysis, we achieved three posteriors associated with three drug conditions, each group posterior included samples of the estimated M-ratio for the corresponding group (Fig 3B). Each subject’s metacognitive efficiencies across the three conditions $M_{Pr,s}$, $M_{Pl,s}$, and $M_{L,s}$ are defined as samples drawn from a multivariate Gaussian distribution:

Given the log-transformed variables, the joint distribution is:


$$\begin{array}{c} \left[\begin{array}{c} \log(M_{Pr,s})\\ \log(M_{Pl,s})\\ \log(M_{L,s}) \end{array}\right]\sim N\left(\left[\begin{array}{c} \mu_{M_{Pr}}\\ \mu_{M_{Pl}}\\ \mu_{M_{L}} \end{array}\right],\left[\begin{array}{ccc} \sigma_{M_{Pr}}^{2} & \rho_{Pr,Pl}\sigma_{M_{Pr}}\sigma_{M_{Pl}} & \rho_{Pr,L}\sigma_{M_{Pr}}\sigma_{M_{L}}\\ \rho_{Pr,Pl}\sigma_{M_{Pr}}\sigma_{M_{Pl}} & \sigma_{M_{Pl}}^{2} & \rho_{Pl,L}\sigma_{M_{Pl}}\sigma_{M_{L}}\\ \rho_{Pr,L}\sigma_{M_{Pr}}\sigma_{M_{L}} & \rho_{Pl,L}\sigma_{M_{Pl}}\sigma_{M_{L}} & \sigma_{M_{L}}^{2} \end{array}\right]\right) \end{array}$$ (3)

Priors are defined as:


$$\begin{array}{llll} \mu_{M_{Pr}},\mu_{M_{Pl}},\mu_{M_{L}} & \sim N(0,1)\; & & \; \end{array}$$

which are average meta-cognitive efficiency in Pr(Propranolol), Pl(placebo) and L(LDopa) conditions. The three standard errors are also associated with three conditions and each was estimated across subjects in the associated condition of task.


$$\begin{array}{llll} \sigma_{M_{Pr}},\sigma_{M_{Pl}},\sigma_{M_{L}} & \sim \text{InvSqrtGamma}(0.001,0.001),\; & & \; \end{array}$$

The correlation between each two conditions, within-subject study, was represented as;


$$\begin{array}{llll} \rho_{Pr,Pl},\rho_{Pr,L},\rho_{Pl,L} & \sim \text{Uniform}(-1,1).\; & & \; \end{array}$$

In addition, the Gelman-Rubin statistic, serving as a measure of convergence for the Hmeta method, remained below 1.1 for all estimations of M-ratio in group-level analyses $\hat{R}$ = 1 . 026, 1 . 019, 1 . 082 for Propranolol, placebo and L-DOPA conditions in-order). We report the number of samples from group posterior which was greater or less between each pair of conditions. At the individual level analysis, the Hmeta package provides samples associated with the posterior distributions for individuals, and the averages of these samples were used as the estimates of the meta-cognitive abilities of each individual under the three drug conditions (Fig 3C). The $\hat{R}$ values for individual estimations of M-ratio ranged from  [ 1 , 1 . 14 ]  (mean  = 1 . 011, sd  = 0 . 008) in propranolol condition,  [ 1 . 019 , 1 . 158 ]  (mean  = 1 . 051, sd  = 0 . 031) in placebo condition and  [ 1 , 1 . 035 ]  (mean  = 1 . 033, sd  = 0 . 031) in L-DOPA condition.

In the final step, we randomly selected 30 samples from each distribution of M-ratio across the three drug conditions and compared them using the Wilcoxon rank-sum test. This comparison was repeated 1,000 times (Fig E in S1), analyzing both the distribution of p-values to determine whether 95% of them were below 0.05.

**Two-outcome task.** For the two-outcome task, we applied Logistic mixed effect regression to measure MF/MB contributions to choice and also the interaction between confidence and MF/MB behavior. The regressors are described in the Results section. The subjects were treated as random effects, so we estimated MF/MB contributions for each subject in three drug conditions. The linear mixed-effects model (using lmer in R) was applied to compare results across drug conditions along with non-parametric Wilcoxon rank sum posthoc tests to compare the effect between each pair of conditions.

We also fitted the hybrid reinforcement model described by Moran et al. [27,42,43] to the behavior of subjects in three drug conditions. In order to shed light on the discrepancies in the findings between the regression analysis on our empirical data in two-outcome task and the results from fitting the mentioned RL model, we simulated the behavior of the hybrid model according to parameters of each subject in each drug condition 1000 times. We performed the mixed effect regression analysis for each run of simulation in each drug condition and we averaged the mixed effects (extracted from regressions) for each subject in each drug condition across all simulations. Thus, we could assess the MB contribution for each subject in every drug condition with behavior coming from the RL model but the assessment from the regression analysis.

## Supporting information

S1 Text Supplementary information is available for this paper.
**Table A.** Demographic and experimental characteristics of participants.
**Table B. The results from questionnaires.** The results of the two questionnaires used in this experiment, PANAS and MCQ-30 are provided.
**Box A:** Influence of drug conditions on Blood Pressure and Heart Rate.
**Box B:** Regression model for Two-outcome task.
**Box C:** The linear mixed effect analysis vs. Repeated Measure Anova.
**Box D:** Computational Modelling for Two-outcome task.
**Fig A. The parameters and fitting score from computational modeling.** The hybrid model was fitted to each drug condition and each subject. The *α* is learning rate, Wmb and Wmf are MB and MF weights, Forget parameter represented the decrease of qvalue for inexperienced options. The Pr and Pz were perseveration parameters. The achieved parameters and fitting score were not significantly different between our drug conditions. The “pro” is abbreviation for propranolol, “dop” for L-DOPA (dopamine) and “plac” for placebo.
**Fig B. Interaction between confidence and MB contribution.** A) Confidence was dichotomized into high and low, relative to the average confidence for each subject. The interaction between confidence and MB contribution was significantly higher in the L-DOPA condition compared to the placebo condition. B) Confidence was analyzed on a continuous scale. Although the effect of L-DOPA was significantly higher compared to Propranolol, it showed a similar trend to the placebo, although not significantly so.
**Fig C. MF and MB contributions in simulated behavior.** We found no significant difference in MB/MF contributions across the three drug conditions. It is important to note that the data used for analysis was simulated behavior(average across 1000 iterations) generated using parameters obtained from computational modeling.
**Fig D. Influence of Drug Conditions on Blood Pressure and Heart Rate.** A) The drug conditions did not significantly influence blood pressure. B) The propranolol condition significantly decreased heart rate relative to both the placebo and L-DOPA conditions.
**Fig E. Comparison between random samples of M-ratio distributions in three drug conditions.** A) The random samples from the M-ratio distribution in Propranolol condition were smaller than the ones in the placebo condition. B) The distribution of p-values, 99 . 6*%* of p-values were less than 0.05. C, D) It was also the case for comparison of propranolol relative to L-DOPA, all the p-values were less than 0.05. E, F) In comparison of L-DOPA condition relative to placebo, just 55 . 5*%* of p-values were lower than 0.05.
**Fig F. Dominant random-walks.** As explained in the main draft, in 69 out of the 90 sessions of our experiment, two random walks were randomly associated with the drug conditions. The starting points were counterbalanced across these two random walks, and the probabilities of reward associated with vegetables did not remain consistently high or low for most of the trial.
**Fig G. Comparison between the influence of two drug conditions.** The red/blue violins show the differences between the Propranolol/L-DOPA and placebo conditions. A) Meta-cognitive ability from individual estimates (Fig 2C of the main draft). B, C) Model-free and Model-based contributions (Fig 3C and 3G). D, E) Interaction between confidence and MF/MB contributions (Fig 3D and 3H).
**Fig H. Correction of M-ratio between two methods of fitting.** We estimated M-ratio according to the classic method [4]. Despite differences between the two methods, we found a significant positive correlation in the placebo condition (B). However, no correlation was observed in the propranolol and L-DOPA conditions (A and C). (PDF)
