# Supplementary material for: Noradrenergic and Dopaminergic modulation of meta-cognition and meta-control
Source: PLoS Comput Biol. 2025 Feb 26;21(2):e1012675. doi: 10.1371/journal.pcbi.1012675 (PMC11903042; doi:10.1371/journal.pcbi.1012675)
Supplement: S1 Text — Supplementary information is available for this paper. Table A. Demographic and experimental characteristics of participants. Table B. The results from questionnaires. The results of the two questionnaires used in this experiment, PANAS and MCQ-30 are provided. Box A: Influence of drug conditions on Blood Pressure and Heart Rate. Box B: Regression model for Two-outcome task. Box C: The linear mixed effect analysis vs. Repeated Measure Anova. Box D: Computational Modelling for Two-outcome task. Fig A. The parameters and fitting score from computational modeling. The hybrid model was fitted to each drug condition and each subject. The α is learning rate, Wmb and Wmf are MB and MF weights, Forget parameter represented the decrease of qvalue for inexperienced options. The Pr and Pz were perseveration parameters. The achieved parameters and fitting score were not significantly different between our drug conditions. The “pro” is abbreviation for propranolol, “dop” for L-DOPA (dopamine) and “plac” for placebo. Fig B. Interaction between confidence and MB contribution. A) Confidence was dichotomized into high and low, relative to the average confidence for each subject. The interaction between confidence and MB contribution was significantly higher in the L-DOPA condition compared to the placebo condition. B) Confidence was analyzed on a continuous scale. Although the effect of L-DOPA was significantly higher compared to Propranolol, it showed a similar trend to the placebo, although not significantly so. Fig C. MF and MB contributions in simulated behavior. We found no significant difference in MB/MF contributions across the three drug conditions. It is important to note that the data used for analysis was simulated behavior(average across 1000 iterations) generated using parameters obtained from computational modeling. Fig D. Influence of Drug Conditions on Blood Pressure and Heart Rate. A) The drug conditions did not significantly influence blood pressure. B) The propran [file pcbi.1012675.s001.pdf]

Supplementary Information

immediate

January 17, 2025

S1 Text. Supplementary information.

N=30	Characteristic	Measure	Mean/Count (std)	Min-Max	range
**Demographics**	Gender	Men/Women	14/16	-	-
	Age	Years	24.57 (3.52)	18-32	18-32
	Height	Centimeters	169.18 (9.58)	150.5-184.25	-
	Weight	Kilograms	67.49 (10.04)	46.7-99.5	-
**Experimental parameters**	drugs' order	pro-plac-dop/dop-pro-plac/ dop-plac-pro/plac-pro-dop/ plac-dop-pro/pro-dop-plac	6/6/5/5/4/4	-	-

Table A: Demographic and experimental characteristics of participants. The "pro" is abbreviation for propranolol, "dop" for L-DOPA (dopamine) and "plac" for placebo.

N=30	Drug	Characteristic	Turn	Mean (std)
**PANAS**	Placebo	Positive Affect	first	29.83 (6.09)
			second	29.07 (6.96)
		Negative Affect	first	18.03 (6.79)
			second	17.23 (7.53)
	L-DOPA	Positive Affect	first	32.17 (7.37)
			second	31.27 (7.87)
		Negative Affect	first	17.37 (5.15)
			second	16.23 (5.35)
	Propranol	Positive Affect	first	32.17 (6.79)
			second	31.03 (7.67)
		Negative Affect	first	16.53 (5.47)
			second	16.17 (5.62)
**MCQ-30**	Placebo	(Lack of) Cognitive Confidence	-	10.53 (3.68)
		Positive Beliefs about Worry	-	12.7 (3.77)
		Cognitive Self-Consciousness	-	17.47 (3.70)
		Negative Beliefs about Uncontrolabilty and Danger	-	11.67 (3.67)
		need to control thoughts	-	12.57 (4.02)
	L-DOPA	(Lack of) Cognitive Confidence	-	10.70 (3.61)
		Positive Beliefs about Worry	-	12.86 (3.84)
		Cognitive Self-Consciousness	-	17.17 (4.07)
		Negative Beliefs about Uncontrolabilty and Danger	-	11.79 (3.38)
		need to control thoughts	-	12.52 (4.01)
	Proponalol	(Lack of) Cognitive Confidence	-	10.70 (3.73)
		Positive Beliefs about Worry	-	12.97 (4.04)
		Cognitive Self-Consciousness	-	17.73 (3.31)
		Negative Beliefs about Uncontrolabilty and Danger	-	11.83 (3.52)
		need to control thoughts	-	12.73 (3.53)

Table B: The results from questionnaires. The results of the two questionnaires used in this experiment, PANAS and MCQ-30 are provided. The range of scores for both Positive and Negative Affect could be 10-50 and 6-24 for all factors of MCQ-30.

Box A: Influence of drug conditions on Blood Pressure and Heart Rate. Blood pressure (BP) and heart rate (HR) were measured twice per session, once before taking the first capsule and again before starting the first task. The difference between the second and first measurements was used to study the influence of drug conditions on these two estimations.

To estimate BP, we measured systolic and diastolic pressures, which correspond to the pressures when the heart beats and between heartbeats, respectively. Mean

Arterial Pressure (MAP) was then estimated as follows:

$$\text{MAP} = \text{Diastolic BP} + \frac{1}{3}(\text{Systolic BP} - \text{Diastolic BP}) \quad (1)$$

The BP was not significantly different between the three drug conditions (propranolol vs. Placebo: $b = -2.389$, $t(58) = -1.298$, $p = 0.2$, $95\%CI = [-6.466, 1.358]$; L-DOPA vs. Placebo: $b = -2.554$, $t(58) = -1.016$, $p = 0.313$, $95\%CI = [-12.453, -0.863]$)(Figure 4A). The effect of the propranolol condition on HR was significant ($b = -6.658$, $t(58) = -2.301$, $p = 0.02$, $95\%CI = [-12.453, -0.863]$), while the L-DOPA condition did not significantly affect HR ($b = 1.404$, $t(58) = 0.485$, $p = 0.629$, $95\%CI = [-4.391, 7.199]$) (Figure 4B). The posthoc non-parametric test showed lower HR in propranolol relative to both Placebo (W = 565, $p = 0.049$) and L-DOPA (W = 260, $p = 0.012$) conditions; while, there was no significant difference between propranolol and L-DOPA conditions (W = 401, $p = 0.611$). Three subjects were excluded from the analyses for BP, and two were excluded for HR due to the lack of recorded data in one of their sessions.

Box B: Regression model for Two-outcome task.

The equations for the logistic mixed regression models explained under the subtitle "MF and MB contributions to choice", the "Result" section;

$$Repeat_t \sim C_{MF,t-1} + U_{MF,t-1} + (C_{MF,t-1} : U_{MF,t-1}|participant) \quad (2)$$

$$Generalization_t \sim C_{MB,t-1} + Prob_t + (C_{MF,t-1} : Prob_t|participant) \quad (3)$$

Box C: The linear mixed effect analysis vs. Repeated Measure Anova. To assess the significance of drug effects in our study, we used both linear mixed-effects analysis and repeated measures ANOVA. In terms of statistical significance, both methods yielded consistent results across all analyses, except for the effect of drug conditions on the interaction between confidence and MF behavior (Fig 3D). Here, the linear mixed-effects analysis showed a significant effect, whereas the

repeated measures ANOVA did not. Post hoc analysis further indicated that L-DOPA significantly reduced this interaction. We reported the statistics from the linear mixed-effects model, as we considered it more suitable for our data structure, given the high number of random effects (30 subjects).

Box D: Computational Modelling for Two-outcome task.

The developed model was a combined reinforcement learning model to describe each participant's sequence of decisions. This model proposes that decision-making is influenced by both model-based (MB) and model-free (MF) processes. For every individual, the MF process stores a Q^{MF}-value, which is accessed when that individual is up for selection. Post feedback on the rewards obtained from vegetables, these Q^{MF}-values for the selected individual are updated using a prediction error, moderated by a learning coefficient lr:

$$Q^{MF}(\text{individual}) \leftarrow Q^{MF}(\text{individual}) + \text{lr} \cdot (\text{cumulative reward} - Q^{MF}(\text{individual})) \tag{4}$$

In contrast, the MB process retains Q^{MB}-values for each vegetable. When making a choice during the trials, the Q^{MB}-value for each presented individual is derived from the underlying transition probabilities:

$$Q^{MB}(\text{individual}) = Q^{MB}(\text{veg1}) + Q^{MB}(\text{veg2}) \tag{5}$$

After making a choice, the MB process updates the Q^{MB}-values for each vegetable according to its received reward:

$$Q^{MB}(\text{veg}) = Q^{MB}(\text{veg}) + \text{lr} \cdot (\text{reward} - Q^{MB}(\text{veg})) \tag{6}$$

Furthermore, the Q^{MF}-values for the three unselected individuals and the Q^{MB}-values for the unobserved vegetables are attenuated by a decay factor f (another model parameter) as follows:

$$Q^{MF}(\text{individual}) = (1 - \text{f}) \cdot Q^{MF}(\text{individual}) \tag{7}$$

$$Q^{MB}(\text{veg}) = (1 - \text{f}) \cdot Q^{MB}(\text{veg}) \tag{8}$$

Additionally, our framework assumes an incremental perseveration effect for selected individuals. After each trial, the perseveration values for each individual are updated thus:

$$P(\text{individual}) \leftarrow (1 - \text{pr}) \cdot P(\text{individual}) + \text{pr} \cdot \mathbb{I}_{\text{individual=selected}} \tag{9}$$

Here, $\mathbb{I}_{\text{individual=selected}}$ denotes the indicator function that is 1 when the individual is selected, and pr is the rate of perseveration, a free parameter of the model.

When a decision is to be made, a combined Q-value for each available individual is computed:

$$Q_{\text{aggregate}}(\text{individual}) = w_{MB} \cdot Q^{MB}(\text{individual}) + (1 - w_{MB}) \cdot Q^{MF}(\text{individual}) + pz \cdot P(\text{individual}) \tag{10}$$

Here, w_{MB} is a freely estimated parameter reflecting the influence of the MB system (with $1 - w_{MB}$ indicating the MF system's influence), and pz is the perseverance factor. The $Q_{\text{aggregate}}$-values for the duo of presented individuals are then input into a softmax decision rule, controlled by a positive parameter β, affecting the likelihood of selection:

$$\text{Prob}(\text{individual}) = \frac{e^{\beta \cdot Q_{\text{aggregate}}(\text{individual})}}{e^{\beta \cdot [Q_{\text{aggregate}}(\text{individual}) + Q_{\text{aggregate}}(\text{other individual})]}} \tag{11}$$

The Q^{MF}-values start at 1 for each individual, while the Q^{MB}-values for each vegetable initiate at 0.5 at the beginning of each experimental segment.

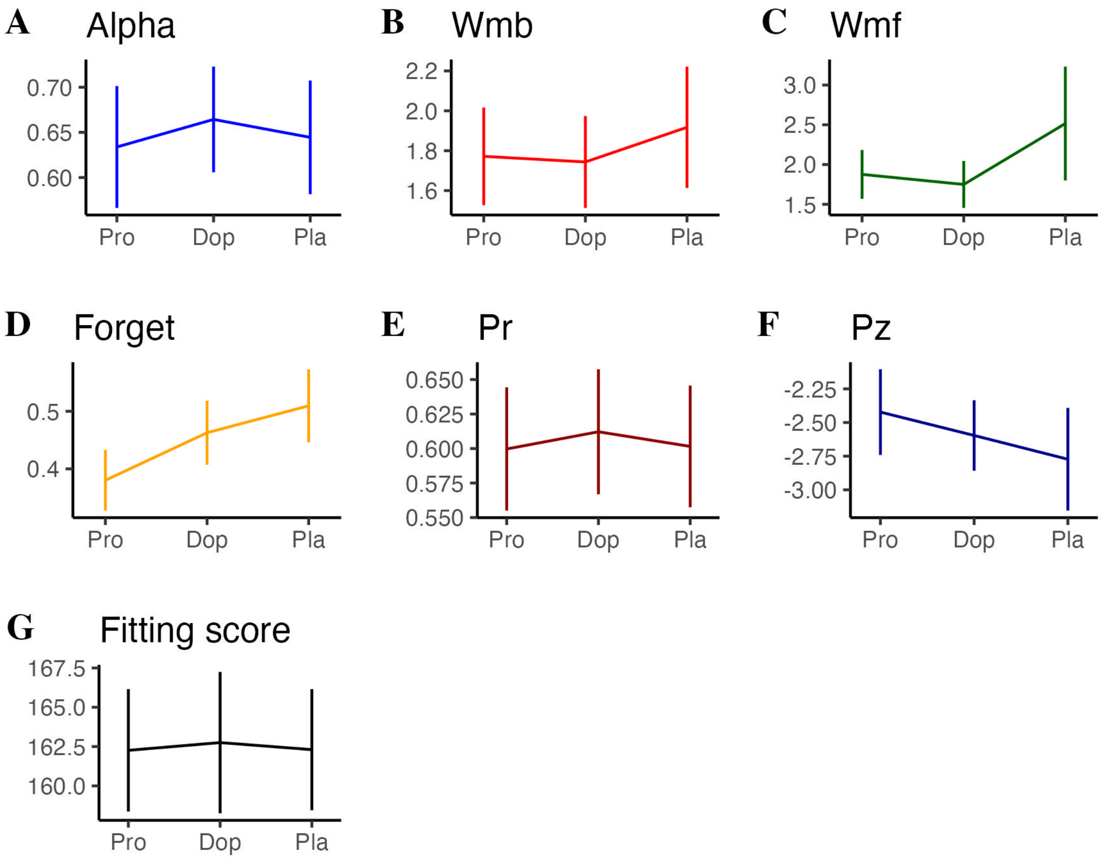

Figure A: The parameters and fitting score from computational modeling. The hybrid model was fitted to each drug condition and each subject. The α is learning rate, Wmb and Wmf are MB and MF weights, Forget parameter represented the decrease of qvalue for inexperienced options. The Pr and Pz were perseveration parameters. The achieved parameters and fitting score were not significantly different between our drug conditions. The "pro" is abbreviation for propranolol, "dop" for L-DOPA (dopamine) and "plac" for placebo.

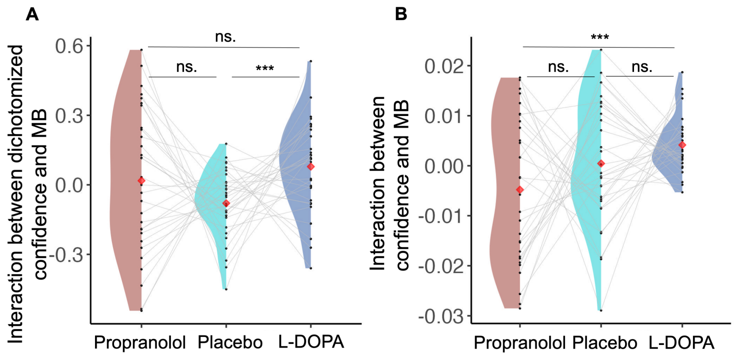

Figure B: Interaction between confidence and MB contribution. A) Confidence was dichotomized into high and low, relative to the average confidence for each subject. The interaction between confidence and MB contribution was significantly higher in the L-DOPA condition compared to the Placebo condition. B) Confidence was analyzed on a continuous scale. Although the effect of L-DOPA was significantly higher compared to Propranolol, it showed a similar trend to the Placebo, although not significantly so.

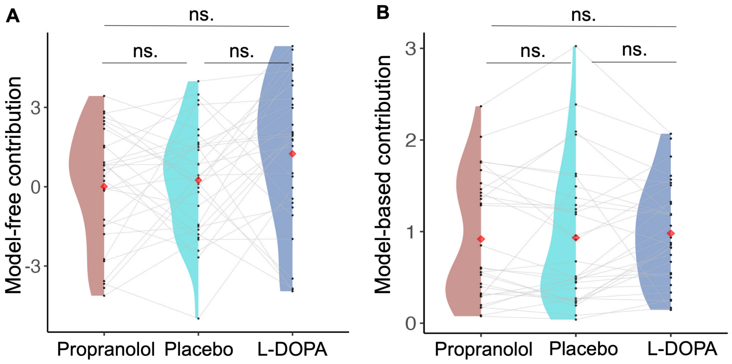

Figure C: MF and MB contributions in simulated behavior. We found no significant difference in MB/MF contributions across the three drug conditions. It is important to note that the data used for analysis was simulated behavior(average across 1000 iterations) generated using parameters obtained from computational modeling.

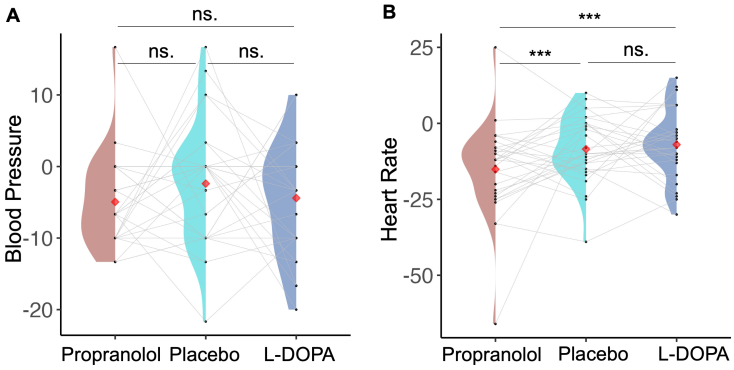

Figure D: Influence of Drug Conditions on Blood Pressure and Heart Rate. A) The drug conditions did not significantly influence blood pressure. B) The propranolol condition significantly decreased heart rate relative to both the placebo and L-DOPA conditions.

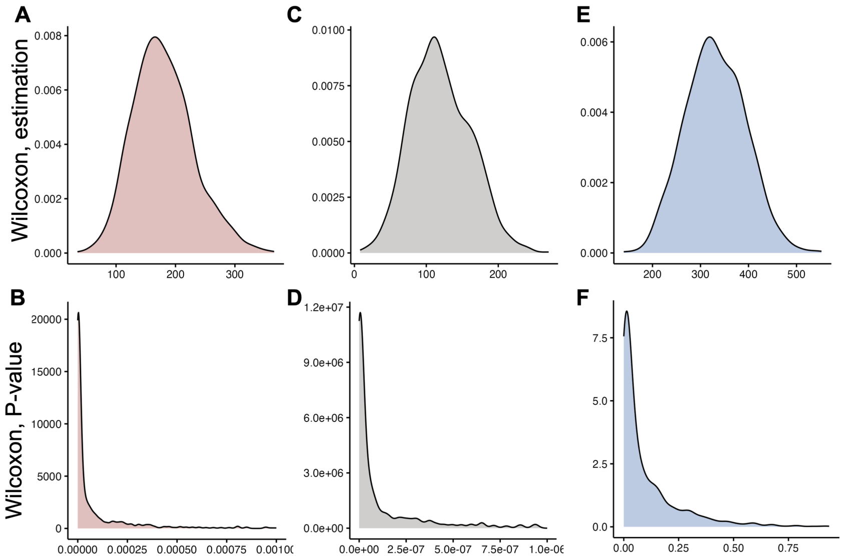

Figure E: Comparison between random samples of M-ratio distributions in three drug conditions. A) The random samples from the M-ratio distribution in Propranolol condition were smaller than the ones in the Placebo condition. B) The distribution of p-values, 99.6% of p-values were less than 0.05. C, D) It was also the case for comparison of propranolol relative to L-DOPA, all the p-values were less than 0.05. E, F) In comparison of L-DOPA condition relative to placebo, just 55.5% of p-values were lower than 0.05.

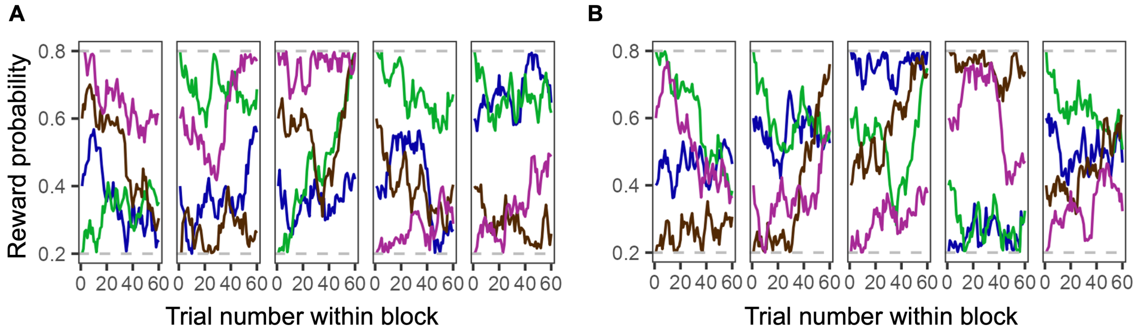

Figure F: Dominant random-walks. As explained in the main draft, in 69 out of the 90 sessions of our experiment, two random walks (as presented in the Supplementary Materials) were randomly associated with the drug conditions. The starting points were counterbalanced across these two random walks, and the probabilities of reward associated with vegetables did not remain consistently high or low for most of the trial.

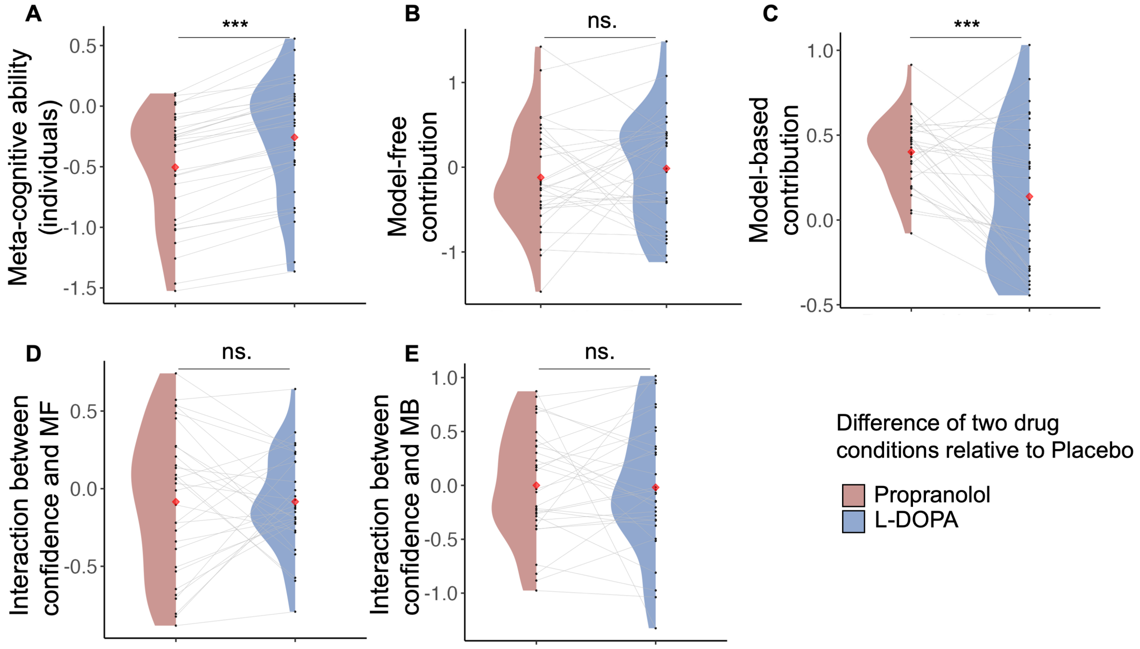

Figure G: Comparison between the influence of two drug conditions. The red/blue violins show the differences between the Propranolol/L-DOPA and placebo conditions. A) Meta-cognitive ability from individual estimates (Figure 2C of the main draft). B, C) Model-free and Model-based contributions (Figures 3C, G). D, E) Interaction between confidence and MF/MB contributions (Figures 3D, H).

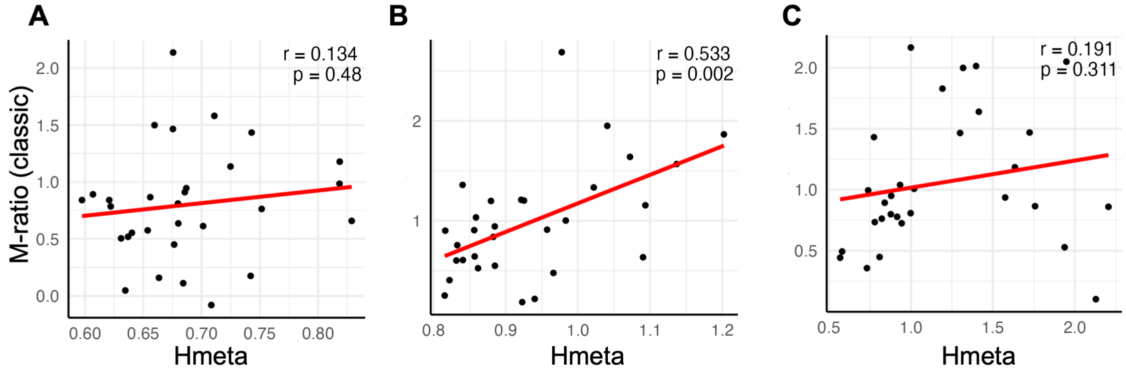

Figure H: Correction of M-ratio between two methods of fitting. We estimated M-ratio according to the classic method. Despite differences between the two methods, we found a significant positive correlation in the placebo condition (B). However, no correlation was observed in the propranolol and L-DOPA conditions (A & C)
